# Comparison of health-related quality of life between the Han and Yi ethnicity elderly in the Yi autonomous areas of Yunnan Province

**DOI:** 10.1186/s12877-019-1257-1

**Published:** 2019-11-25

**Authors:** Lingyun Ran, Hongqian Kong, Mengqi Du, Jianhui He, Qiong Zhong, Yuanyuan Ran, Yanping Si, Jiang Zhang, Cheng Yao, Huineng Luo, Qin Ye

**Affiliations:** 1 Nursing School of Kunming Medical University, Kunming, Yunnan China; 2 Qujing Center for Disease Control and Prevention, Kunming, Yunnan China; 3 Steinhardt School of New York University, New York, USA; 4 Public Health School of Kunming Medical University, Kunming, China; 5 The Sixth Affiliated Hospital of Kunming Medical University, Kunming, Yunnan Province China; 6 Hunan University of Finance and Economics, Changsha, China; 7 The Third Affiliated Hospital of Kunming Medical University, Kunming, Yunnan China; 8 The First Affiliated Hospital of Kunming Medical University, Kunming, Yunnan China

**Keywords:** Health-related quality of life, Elderly, Yi ethnic minority, ADL, IADL

## Abstract

**Background:**

The purpose of this research was to assess the health-related quality of life (HRQOL) and functional abilities of Yi and Han elderly who resided in Yi Autonomous prefecture or Counties in Yunnan Province, as well as to compare their differences in HRQOL, functional abilities and other factors.

**Methods:**

A total of 1636 older subjects, which included 863 Han and 773 Yi, were recruited from 10 Yi Autonomous regions. Their HRQOL and functional capabilities were assessed by the MOS 36-Item Short Form Health Survey (SF-36), activities of daily living (ADL), and instrumental activities of daily living (IADL) scales.

**Results:**

The Han elderly performed better in every domain of SF-36 than the Yi elderly. Both of the two ethnic groups could perform their ADL independently but the Yi elderly showed greater dependency in IADL abilities. The HRQOL was positively associated with their ADL, IADL, and education levels. Moreover, age, health insurance status, and living arrangement were negatively correlated with HRQOL.

**Conclusion:**

The HRQOL and IADL capabilities of the Han elderly were higher than that of the Yi counterparts in the Yi Autonomous regions. The HRQOL of both the two ethnic groups was positively connected with ADL, IADL abilities as well as education levels, whereas it was negatively correlated with age and health insurance. The elderly-care policy on the Yi autonomous areas should focus more on the HRQOL, ADL improvement, education background, age needs, and health insurance, etc.

## Background

Yunnan province, located in southwestern China, is bounded by Laos, Myanmar, and Vietnam [1]. It is also one of the least developed provinces of the country in terms of economy, science, technology or social services. There are 26 ethnic groups in Yunnan, including 25 ethnic minorities and the national majority Han people. Yunnan is characterized by its scattered spatial distribution of the population, with small settlements of different ethnic groups dotted across the province. In total, there are 8 minority autonomous prefectures and 29 minority autonomous counties in Yunnan, which makes the province having the largest number of autonomous prefectures and counties in China [2].

The population of Yunnan is about 45.96 million, with the 25 minority groups of 15.33 million, or roughly accounting for one-third of the total population [3]. The Yi is the most populous minority group in Yunnan, with approximately 5.41million people [4]. Compared with Han people which are the majority in Yunnan, most ethnic Yi people and other minorities live in relatively adverse situations [5]. Ethnic Yi people can be seen all over the whole province, particularly in Chuxiong and Honghe Yi autonomous prefectures where they’re most concentrated [6].

It has been estimated that the elderly population is about 5.7 million in Yunnan province, accounting for 11.95% of the whole population. This number will grow to 12.4% of the whole population in 2020 [7], which means that Yunnan is facing a massive challenge of aging. There is no official statistic data on the elderly population of Yi in Yunnan, but some researchers reported that the life expectancy of the Yi ethnicity is much shorter than that of the Han ethnicity. It has been revealed that elderly people, aged 65 or above, constitute only 4.97% of the whole population of Yi ethnicity [8], which is much lower than the Han people.

Followed aging, the elderly often encounter health problems, mental illness, and decline in function. It is an essential task for healthcare providers to concentrate on retaining or even improving life quality of the elderly population [9]. The World Health Organization defined Quality of life (QOL) as individuals’ perception of their position in life in the context of culture and value systems in which they live and in relation to their goals, expectations, standards and concerns” [10]. One of the essential issues of the elderly is the health-related quality of life (HRQOL) that comprises physical and mental health and their associations with functional abilities, illnesses risks, socioeconomic conditions and social support [11]. The HRQOL is associated with chronic diseases such as hypertension, diabetes, cancer, and risk factors such as body mass index, smoking habits, and physical activities [12]. HRQOL measurement examines the impact of changes in health status and points out further considerations for clinical management and policy-making [13]. Multiple instruments are applied to assess HRQOL such as Medical Outcomes Study Short Forms (SF-12 and SF-36), the Sickness Impact Profile, and the Quality of Well-Being Scale, World Health Organization Quality of Life BREF (WHOQOL-BREF) [14, 15]. These measurements have been widely used in both the community and hospital settings.

Another focus on the elderly is to conduct a functional assessment in order to identify an older adult’s ability to perform self-care, self-maintenance, and physical activities to plan for appropriate nursing interventions. Tools used to assess functional ability tend to address self-care (basic activities of daily living or ADLs), higher-level activities necessary to living independently in the community (instrumental activities of daily living or IADls), or highest level activities (advance activities of daily living or AADLs) [16].

A few studies have focused on the HRQOL of elderly Chinese. Elderly people in rural China almost always reside in a less privileged environment with poorer educational and social support compared with their urban counterparts [17]. HRQOL of the rural elderly is one of the essential topics in our country as the aging of population becomes a social problem. Liang and Wu reported that HRQOL among the empty-nest elderly in rural China was low, with men scoring higher in HRQOL than women. Furthermore, they discovered that older adults with higher degree of education attained higher scores than those with lower education levels [18]. Zhang et al. revealed that the HRQOL of the elderly in neighboring Guangxi province was not ideal, and called on more concern and support to be designed to improve the health of the rural elderly, especially the female, oldest, and Yao groups [19]. Our previous research on elderly Yi found that their HRQOL and functional capacity were much lower compared to the average Yunnan residents or the elderly in other developed areas in China [20]. Since there were no agreed standards of the ADL, IADL, and SF-36 elderly in Yunnan, we decided to compare these items with other studies on the average Yunnan population and older adults in economically developed areas in China. In this research we hope to explore the average level of ADL, IADL, and SF-36 scores in Yunnan elderly population, and the differences between the Yi and Han people based on our previous survey.

Based on previous studies, we assumed that the ADL, IADL, and SF-36 levels of Yi elders were lower than those of Han elders living in the same area. Several factors may also result in the differences in the HRQOL grades between the two ethnicities. This study was designed to assess the HRQOL of the elderly in Yunnan Province and compare the differences of HRQOL between the Han and the Yi people among the Yi autonomous areas.

## Methods

### Sampling methods

This cross-sectional research was conducted among the Han and Yi elderly from the Yi autonomous regions in Yunnan province. The sampling elderly were voluntarily selected from 10 Yi autonomous areas. Han and Yi elderly who were aged 60 or above and willing to join in this survey while living in the desired regions in Yunnan province were included in this study. The exclusion criteria were as follows: unwillingness to attend this study, not residing in Yi autonomous areas, and suffering from mental problems. A total of 1636 elders including 863 Han and 773 Yi were recruited from 10 Yi autonomous areas in Yunnan province. All participants completed the questionnaires read by trained researchers from Kunming Medical University who could speak both Mandarin and Yi language. The protocol of this study was approved by Kunming Medical University and the local government. Verbal and written informed consents from the participants were acquired at the beginning of these interviews.

### Ethical statement

The current study required verbal and written informed consents from the participating elderly and the ethical approval from the Kunming Medical University.

### Measurements

#### HRQOL

SF-36 scale is a valid tool to assess the general health in various population studies. It covers eight domains of HRQOL: physical functioning (PF), role functioning due to physical problems (RP), bodily pain (BP), general health (GH), vitality (VT), social functioning (SF), role functioning due to emotional problems (RE), and mental health (MH). The score of each dimension varies from 0 to 100, or the worst to the best quality of life [21, 22]. The Cronbach α of SF-36 for the present study was 0.896.

#### ADLs

The functional status of the older subjects were evaluated by Activities of Daily Living (ADL) scale that is validated in both hospitals and communities [23]. The ADL scales comprise 3 tools in total to assess the functional decline among the older population. Two of them, which are basic activities of daily living (ADLs) and instrumental activities of daily living (IADLs), have been applied into the current study. The Barthel Index is the ADL tool used in this study that addresses the need for assistance in bathing, eating, dressing, transferring, toileting, and continence. The score of ADL ranges from 0, which indicates “fully dependent”, to 100, which stands for “totally independent” in activities of daily living. More specifically, ADL grades 0–19 imply “entirely dependent”; 20–40 “severely dependent”; 41–60 “moderate dependent”; and 61–99 “mildly dependent” [24].

The instrumental activities of daily living (IADLs) scale comprise the ability of using the telephone, cooking, shopping, doing laundry, housekeeping, managing finances, taking medications, and preparing meals. These functional abilities are considered to be more complex than ADLs and address the elderly’s interactions with the environment and community. The total score of IADL subscale varies from 0 to 8, with the higher grades indicating independent IADL levels [25, 26]. The internal consistency was measured by Cronbach α coefficient, which was 0.955 and 0.739 for these two ADL scales, respectively.

### Other variables

A few variables were designed by the research group in this study. These included gender, ethnicity, education levels, age group, living arrangement, and health insurance status. Education background was classified into four categories: illiterate, primary school, junior high school, and senior high school and above. The sampling elderly population were divided into five age groups, which were 60–64, 65–69, 70–74, 75–79, and 80 years and older. “Living alone” or “Living with family members” was given for the living arrangement choices. “Yes” or “No” choices were offered for the health insurance coverage.

### Statistical analysis

We used Epidata 3.1 to establish the database and Statistical Package for Social Sciences (SPSS) version 22 to analyze the collected data. Means and standard deviations were utilized to express the statistical results of continuous variables, while proportions and frequencies were applied to show categorical variables. t-test was used to indicate the statistical differences between the Han and Yi elderly in ADL, IADL and HRQOL areas. Pearson correlation analysis was used to analyze the association between HRQOL and functional abilities in the sample population. Pearson correlation analysis was also utilized in reasoning the related factors between HRQOL and age, education levels, living arrangements, as well as health insurance situation.

## Results

### Sociodemographic characteristics of participants

A total of 1636 elderly were enrolled in this research and responded to the interviews, which included 863 Han elderly and 773 Yi older subjects. Among them, 758 were male (46.3%) and 878 were female (53.7%), while Han and Yi elderly subjects each accounted for 52.8 and 47.2%, respectively. The overwhelming majority (98.5%) of the participants had medical insurance, and 87.5% of them were living with their family members. Table 1 describes the sociodemographic characteristics of the participating elderly.

**Table 1:** Sociodemographic characteristics of the participating population (*n* = 1636)

	Number (percent, %)
Gender	
Male	758 (46.3)
Female	878 (53.7)
Ethnicity	
Han	863 (52.8)
Yi	773 (47.2)
Education levels	
Illiterate	274 (16.7)
Primary school	599 (36.6)
Middle school	402 (24.6)
High school and above	361 (22.1)
Age-group	
60–64	399 (24.4)
65–69	498 (30.4)
70–74	354 (21.6)
75–79	189 (11.6)
80 years and above	196 (12.50)
Living arrangement	
Living alone	205 (12.5)
Living with family members	1431 (87.5)
Health insurance	
Yes	1612 (98.5)
No	24 (1.5)

### ADL and IADL scores

Table 2 summarizes the ADL and IADL mean scores and standard deviations of the Han and Yi ethnicity elderly. The mean (SD) of the ADL score for the Han elderly was 91.7 (±19.8), while the ADL grades of the Yi elderly stood at 92.3 (±17.4). The result of the IADL mean score of the Han subjects was 5.8 (±1.8), which was significantly higher than that of the Yi elderly.

**Table 2:** ADL and IADL scores differences between the Han and Yi elderly

Ethnicity	ADL scores (mean ± SD)	IADL (mean ± SD)
Han	91.7 ± 19.8	5.8 ± 1.8*
Yi	92.3 ± 17.4	4.8 ± 1.7
*P* value	0.582	0.000

Note: * denotes statistical significance compared with Yi group

### Han and Yi elderly scores in eight domains of the SF-36 scale

**Fig. 1:**
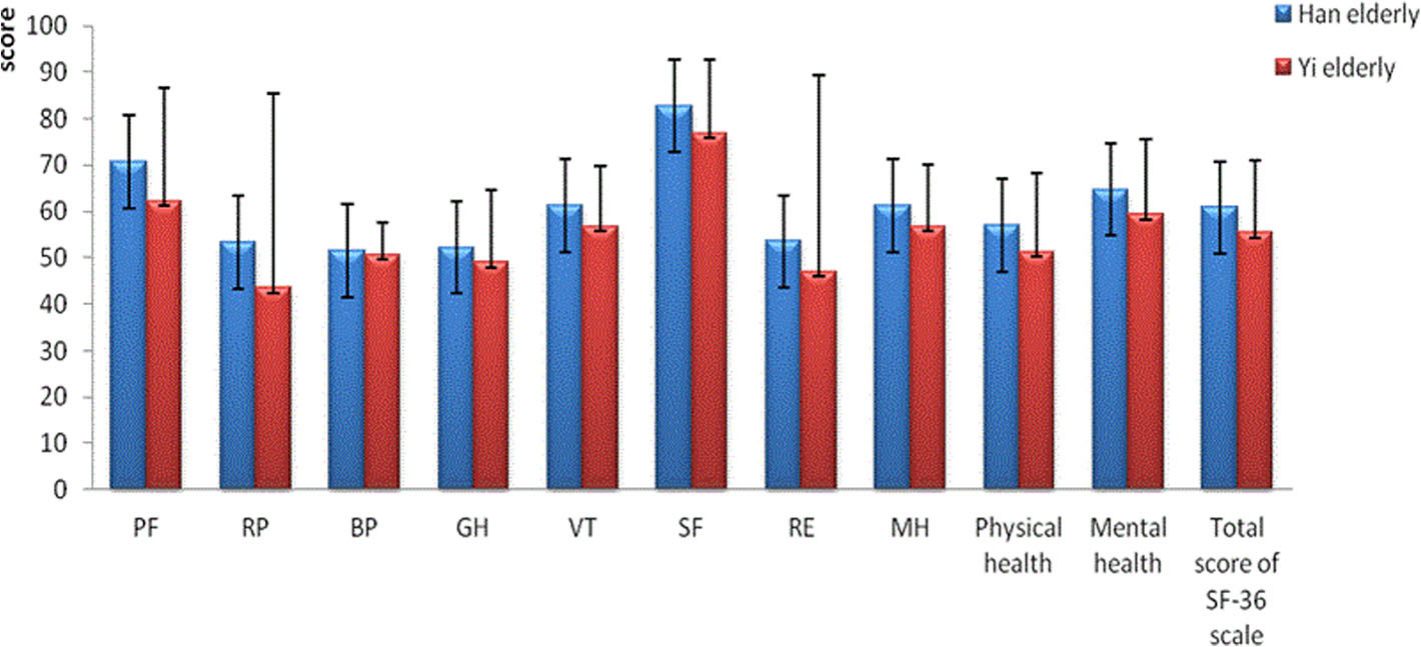
Comparison between Han and Yi elderly subjects on eight domains of SF-36

Figure 1 demonstrated that the overall score of the total sample in the SF-36 scale was 59.5 ± 17.3, indicating that the HRQOL of this population was not ideal. Of the total eight domains, SF received the highest score, followed by PF, VT, MH, RE, GH, BP, and RP. The Han elderly got a higher total grade of 60.8 ± 17.6, compared with the Yi elderly of 55.3 ± 15.6 in SF-36. As can be seen, the Han ethnic group obtained higher values in each dimension of SF-36 than the Yi elderly. Statistical differences were found between Han and Yi elderly subjects in each domain of SF-36 as well as the overall score in SF-36.

### Correlation analysis between HRQOL and ADL, IADL

Pearson correlation analysis was used to analyze the association between HRQOL scores and ADL, IADL levels, as well as sociodemographic factors. Table 3 demonstrated that HRQOL was positively correlated with ADL and IADL, which meant that the more independent the elderly were, the higher their level of health-related quality of life was. The HRQOL was also positively associated with education levels but negatively related to age, health insurance and living arrangement. On the other hand, gender was not related to HRQOL in this population.

**Table 3:** Correlation analysis between HRQOL and correlated factors

Items	ADL	IADL	Education levels	Age	Health insurance	Living arrangement	Gender
	r(p)	r(p)	r(p)	r(p)	r(p)	r(p)	r(p)
HRQOL	0.42 (0.01)	0.27 (0.01)	0.16 (0.00)	−0.24 (0.00)	−0.07 (0.00)	− 0.04 (0.14)	−0.02 (0.52)

## Discussion

The sampling population of this cross-sectional study was conviently recruited from 10 Yi Autonomous regions in Yunnan Province. Actually this research didn’t control the age, gender, education background, living arrangement and health insurance so the article does not compare the above items between Han and Yi ethnicities in Table 1. The HRQOL assessed the functional abilities of the elderly using SF-36, ADL, IADL tools among Han and Yi ethnic elderly subjects from 10 Yi autonomous areas in Yunnan province. This research identified that both the Health-related Quality of Life of the Han and Yi ethnic group elderly was not ideal, although Han elderly had higher scores in SF-36 compared to the Yi elderly. There was no statistical difference in ADL abilities between elderly subjects of the two ethnicities, while aged Yi people attained lower scores in IADL capacities. The ADL, IADL abilities and education levels of the elderly were positively correlated with their HRQOL scores. Those findings indicated that independent ADL and IADL resulted in better HRQOL. Our research also found that age, health insurance and living arrangement were negatively related to HRQOL, meaning that older age, absence of health insurance and living alone contributed to worse HRQOL.

HRQOL comprises physical and mental health, social relationships, and the association between a subject and their environment [26]. Our previous research on elderly Yi ethnic minority in China revealed that education background, ADL, IADL were positively associated with HRQOL, whereas age, chronic diseases, and medication frequency were negatively correlated with HRQOL [20]. Due to the limited sampling size of our former, there was no previous comparison of ADL and IADL scores between the elderly subjects of the two ethnic groups. The current research enlarged the sampling population and supplemented previous limitations. The present study demonstrated that older residents of both the two ethnic groups can mostly perform their ADL items independently, but Yi older subjects showed greater dependency in IADL functioning. Functional dependency has been revealed to be associated with lack of healthcare, isolation, low income, experiencing fall, depression, and frequency of outdoor activities [27]. The IADL items comprise the capabilities of utilizing the telephone, preparing meals, shopping, doing laundry, taking medication, and managing finances, etc. The finding of this research in the Yi and Han elderly was consistent with other studies on associations between HRQOL and ADL, IADL abilities. Education is also an essential factor of HRQOL, which has again been pointed out in other studies [28]. Our study reconfirmed that higher education contributed to better HRQOL. Moreover, older age, absence of health insurance, and living alone are factors to worse HRQOL. Machón et al. also found that health, social and contextual variables were significantly related to HRQOL in independent community-dwelling elderly subjects [29]. The Yunnan locals are still in great demands of basic public health service, although most of them are covered by health insurance. Some researchers suggested that primary health care and public health practitioners need to pay more attention to managing multi-morbidity and preventing chronic diseases to improve the HRQOL [30]. Future elderly care policy should focus on the multidimensional nature of HRQOL, especially the well-being of the minority elderly.

## Conclusion

The HRQOL of the Han elderly was better than that of the Yi ethnic group in the Yi autonomous areas in Yunnan province. The ADL, IADL, and education levels were positively correlated with their HRQOL grades in the sampling population. However, older age, health insurance, and living arrangements contributed to worse HRQOL. The elderly-care policy in the Yi autonomous areas should pay more attention to the HRQOL and its influential factors.

## Data Availability

Data are available from authors on request.

## Abbreviations

ADL: Activities of daily living
HRQOL: Health-related quality of life
IADL: instrumental activities of daily living
WHOQOL-BREF: World Health Organization Quality of Life BREF
